# Validating the socio-spiritual items of the Utrecht Symptom Diary—4 Dimensional: Content and construct validity

**DOI:** 10.1177/02692163251321692

**Published:** 2025-02-28

**Authors:** Tom Lormans, Everlien de Graaf, Frederieke van der Baan, Carlo Leget, Saskia Teunissen

**Affiliations:** 1 Center of Expertise Palliative Care Utrecht, UMC Utrecht, Utrecht, The Netherlands; 2 Department of Medical Oncology, UMC Utrecht, Utrecht, The Netherlands; 3 University of Humanistic Studies, Utrecht, The Netherlands

**Keywords:** Palliative care, patient care, spirituality, social behavior, validation studies, patient reported outcome measures

## Abstract

**Background:**

The Utrecht Symptom Diary—4 Dimensional (USD-4D) is a multidimensional Patient-Reported Outcome Measure to monitor symptoms and needs and increase patients’ self-efficacy. Assessing the content and construct validity of the USD-4D ensures it accurately measures the intended construct and is contextually relevant.

**Aims:**

This study aimed to assess the content and construct validity of the socio-spiritual items of the USD-4D in a population of Dutch patients in the palliative phase of their illness.

**Design:**

A multiple method study was performed consisting of a cross-sectional survey and an observational cohort study.

**Participants:**

The study population consisted of (a) healthcare providers working with patients in the palliative phase and (b) a cohort of patients with a life limiting illness in all settings supplemented by a cohort of hospice patients.

**Results:**

At least 80% of participants positively assessed the items comprehensibility and relevance. About half of the respondents indicated that certain items are missing from the USD-4D. A qualitative analysis of missing topics revealed either topics for monitoring over time or topics underlying the constructs included. For every item, at least 75% of hypotheses were confirmed. One hypothesis for the item “I can let my loved ones go” was rejected.

**Conclusions:**

This study confirmed the content and construct validity on the socio-spiritual items of the USD-4D. Hence, the USD-4D is a validated PROM suitable to be structurally used in clinical palliative care to signal, monitor and to go into dialogue about social and spiritual aspects of patients’ values, wishes, and needs.

**What is already known about the topic?**

- Although many Patient-Reported Outcome Measures (PROM) are validated, not all are suitable to monitor patients’ multidimensional symptoms and needs in day-to-day palliative care.
- The Utrecht Symptom Diary—4 Dimensional (USD-4D) is useful in monitoring these multidimensional symptoms and needs.
- The content validity of the socio-spiritual items of the USD-4D has been positively assessed from the patient’s perspective.

**What this paper adds?**

This paper assesses the content validity of the socio-spiritual items of the USD-4D from the perspective of healthcare providers and the construct validity of these items.

- The USD-4D is clinically feasible for monitoring patients’ multidimensional symptoms and needs and facilitates patient-driven dialogue, fostering holistic patient-centered palliative care.
- After culturally sensitive translation and cross-cultural validation, the USD-4D is also suitable for broader application.

**Implications for practice, theory or policy**

- A PROM does not need an inexhaustible set of items to assess patients’ socio-spiritual needs.
- Healthcare providers need to consider how the USD-4D needs to be properly embedded in clinical care for patients to optimally benefit from using it.
- Using the USD-4D cannot be seen without patient-healthcare provider dialogue on the items and outcomes.

## Introduction

Patients with life-limiting illnesses face multidimensional symptoms and needs that require personalized care to maintain quality of life.^1–3^ Patient-Reported Outcome Measures (PROMs) support symptom management by incorporating patients’ assessments of their symptom severity,^2,3^ alleviating suffering and improving well-being.^4^

The Edmonton Symptom Assessment System (ESAS) is a widely used PROM for physical and psychological symptoms.^5,6^ In the Netherlands, its adapted version, the Utrecht Symptom Diary (USD), fulfills a similar role.^7^ However, the social and spiritual dimensions of care fall outside of the scope of these PROMs and often remain insufficiently addressed in clinical practice.^8,9^

PROMs like the Social Difficulties Inventory (SDI),^10^ Functional Assessment of Chronic Illness Therapy—Spiritual Well-Being (FACIT-Sp),^11^ and Spiritual Needs Assessment for Patients (SNAP)^12^ focus on the social and spiritual dimensions. However, they are time-intensive, lack holistic scope, and are most effective when specific needs are already identified and the social and spiritual dimensions must be explored further.

**Figure 1.**
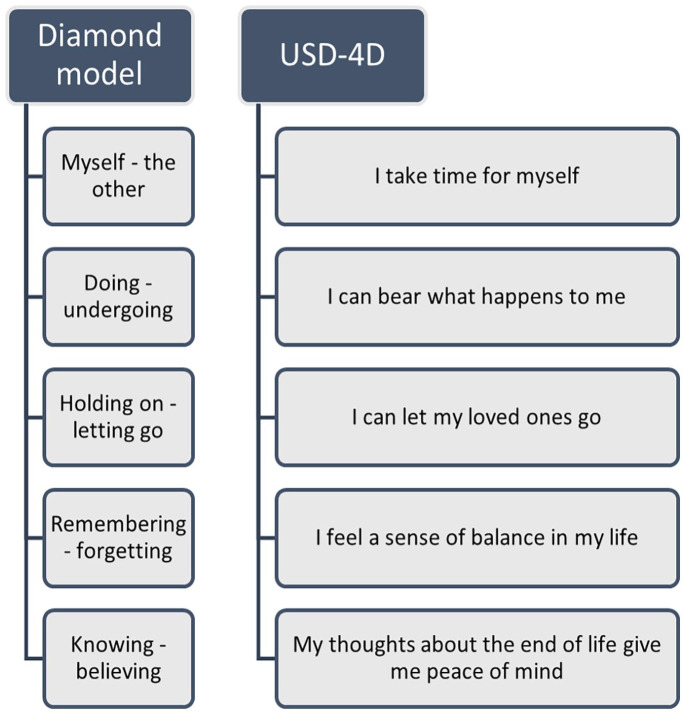
The anthropological polarities of the diamond model and their respective operationalized items in the USD-4D.

To address this gap, the USD was expanded into the Utrecht Symptom Diary—4 Dimensional (USD-4D), incorporating five socio-spiritual items based on the Diamond Model.^13^ The Diamond Model is a validated tool that helps patients and healthcare providers discuss social and spiritual issues.^14,15^ It centers on the concept of “inner space,” a state of mind connecting individuals to their emotions and attitudes. Its five polarities have been adapted into five USD-4D items that reflect social, spiritual, or both dimensions, depending on the patient’s perspective, see Figure 1.^9^ Now, the USD-4D’s 21 items, scored on an 11-point intensity scale, allow patients to prioritize multidimensional symptoms and needs, fostering autonomy and patient-centered care (Appendix 1).

The USD-4D has been implemented in Dutch healthcare, showing feasibility in addressing patients’ symptoms, wishes, and needs.^16^ Its socio-spiritual items were validated by patients for comprehensiveness, relevance, and clarity.^17^ However, content validity from the healthcare providers perspective and construct validity, how well the PROM measures its intended constructs, remain to be evaluated.^18,19^

This study aims to assess the content validity of the USD-4D from healthcare providers’ perspectives and evaluate the construct validity of its socio-spiritual items in Dutch patients facing life-limiting illness.^19^

## Methods

This observational, multi-method study addressed two aims: (1) content validity was assessed via a cross-sectional online survey of healthcare providers (October 2020–August 2021); and (2) construct validity was evaluated in 2022 through a cohort study using the USD-4D, completed by patients with a life expectancy under 1 year.

The study adhered to the Checklist for Reporting Results of Internet E-Surveys (CHERRIES)^20^ as well as the Strengthening the Reporting of Observational Studies in Epidemiology (STROBE) guidelines^21^ to ensure reporting rigor.

### Population and setting

#### Content validity

A convenience sample of healthcare providers working in palliative care were invited to participate based on accessibility. The sample included nurses, physicians, social workers, chaplains, and volunteers aged 18 or older across various settings (hospital, nursing home, home, hospice). Participants were selected through palliative care organizations. Prior experience with the USD-4D was not required. The goal was to recruit at least 15 healthcare providers per discipline and setting for a diverse, representative sample.

#### Construct validity

Two patient cohorts in the palliative phase were included:

*SYMPAL Cohort*: Established in 2009, this hospice-based cohort includes patients aged 18 or older with an estimated life expectancy under 3 months. Participants had self-completed at least one USD-4D during their admission.
*MuST-PC Cohort*: Includes patients aged 18 or older with an estimated life expectancy under 1 year, assessed using the “surprise question” (“Would I be surprised if this patient died in the next 12 months?” with a “No” response). Participants self-completed a USD-4D for this study.

### Ethical issues

This study followed the Declaration of Helsinki and General Data Protection Regulation to ensure ethical conduct and data protection. The UMC Utrecht institutional review board determined the research was exempt from the WMO (decisions 18-499/C, July 2018, and 19-602/C, 2019). Similarly, the University Medical Center Groningen concluded the MuST-PC cohort study was outside WMO’s scope (decision 2018/307).

### Study outcome

#### Content validity

The study outcome was the content validity of the socio-spiritual items of the USD-4D as perceived by healthcare providers (Table 1).

**Table 1.** Aspects of content validity.^22^.

Aspect of content validity	Definition
Comprehensibility	The items should be understood and interpreted by HCPs as intended
Relevance	All items should be relevant for the construct of interest within a specific population and context of use
Comprehensiveness	No key aspects of the construct should be missing

#### Construct validity

The outcome of this study was the assessment of the construct validity of the socio-spiritual items within the USD-4D. Each of the five socio-spiritual items was treated as a distinct construct, and consequently, the construct validity of each item was evaluated through hypothesis testing.^19^ At the basis of the formulation of the hypotheses laid the concept of “total pain”: “the suffering that encompasses all of a person’s physical, psychological, social, and spiritual struggles.”^23^ In other words, social and spiritual well-being affects and is affected by physical and psychological symptoms and needs and vice.^24–28^

**Figure 2.**
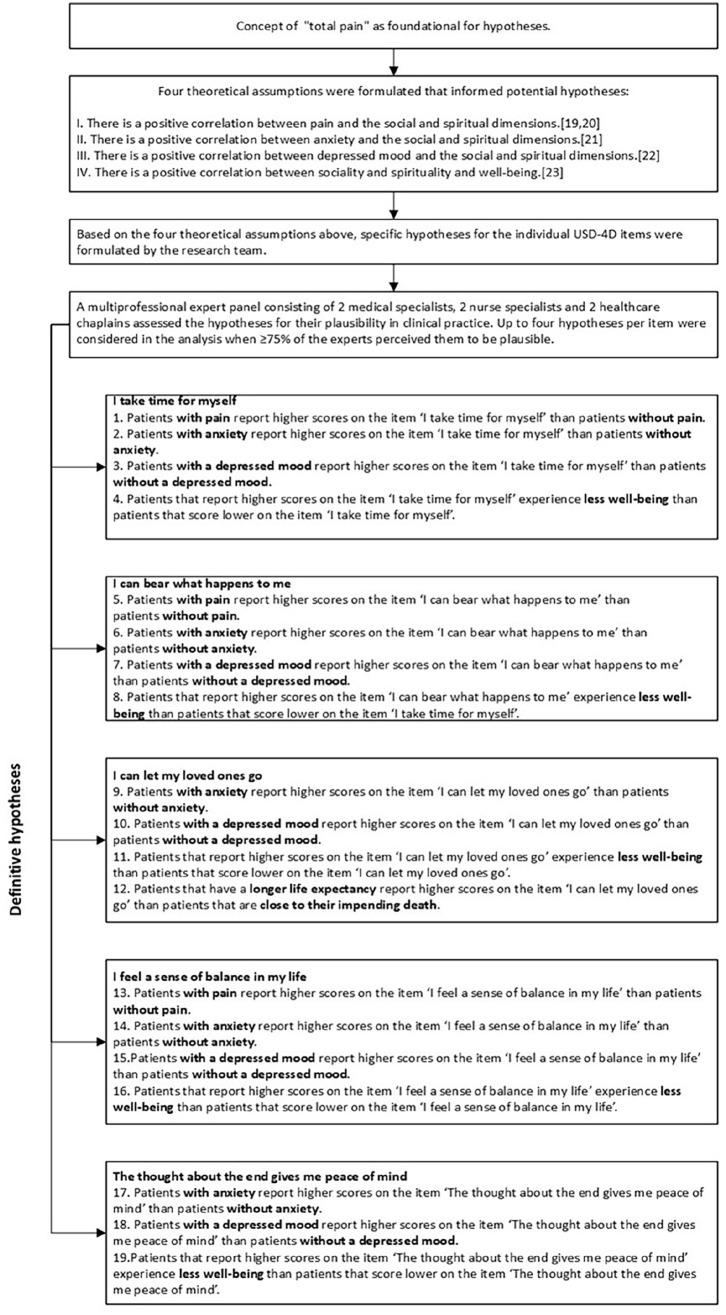
Flowchart of hypotheses-development.

For all items except one, four hypotheses were developed. For the final item, “The thought about the end gives me peace of mind,” three hypotheses were formulated (Figure 2). Following the COSMIN methodology, construct validity for an item was confirmed when at least 75% of the formulated hypotheses for that item were confirmed. For the item with only three hypotheses, a cut-off of 66% was applied to establish construct validity.^19^

### Data collection

#### Content validity

Data were collected using a Survey Monkey-based survey designed for this study. The survey included 22 items evaluating the comprehensibility, relevance, and comprehensiveness of the USD-4D socio-spiritual items, rated on a four-point Likert scale (“totally disagree” to “totally agree”). An open-ended question invited suggestions for missing items, and additional questions captured respondent characteristics. The survey link was openly shared, ensuring broad access while maintaining participant anonymity by collecting no identifiable information.

**Figure 3.**
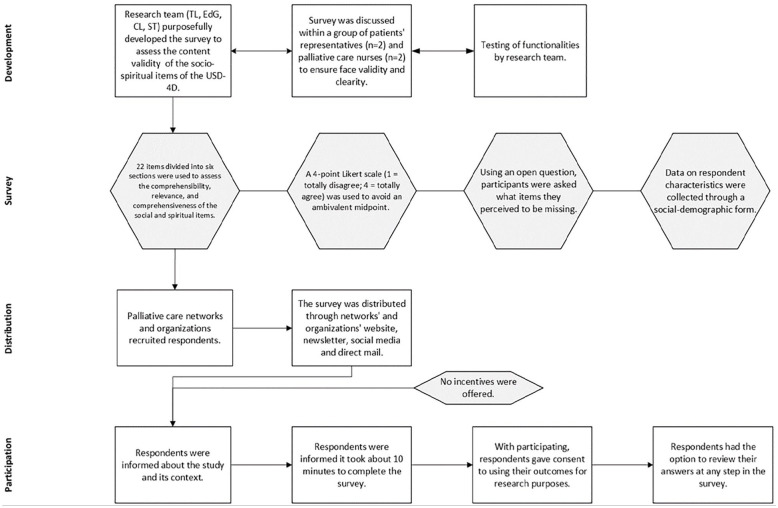
Development, distribution, and participation process of the survey.

Figure 3 outlines the survey’s development, distribution, and participation process.

#### Construct validity

For all hypotheses except hypothesis 12, only the first completed USD-4D per patient was analyzed. If multiple USD-4Ds were available, only the earliest was selected. For hypothesis 12, paired USD-4D scores were created for each patient, consisting of the first completed USD-4D and one completed within 2 weeks before death.

### Statistical analysis

#### Content validity

Data from the online survey program were automatically imported into IBM SPSS 26 for analysis.^29^ Descriptive statistics (frequencies, mean, SD, and range) summarized respondents’ characteristics. Missing items identified by healthcare providers were analyzed using content analysis.

The Item-level Content Validity Index (I-CVI) was calculated for each item. Responses were dichotomized: “agree and totally agree” (scores 3 and 4) or not (scores 1 and 2). The I-CVI was then computed as the proportion of positive responses (“agree” and “totally agree”) out of the total responses, with values >0.8 considered excellent.^22^

#### Construct validity

Before analysis, data from the two cohorts were reorganized and recoded into similar variables. Age was categorized as an ordinal variable, consistent with the MuST-PC cohort.

Most hypotheses and theoretical assumptions focused on the absence versus prevalence of symptoms. To assess the alignment of USD-4D scores with these hypotheses, items were dichotomized into “no symptoms” (score = 0) and “severe symptoms” (score ⩾6). However, “well-being” was treated as a continuous variable, in line with previous research, and not dichotomized.^30^

Statistical tests included:

- *Chi-squared test*: For hypotheses on dichotomized items (e.g., pain, anxiety, depressed mood).
- *Mann–Whitney U test*: For hypotheses on “well-being,” as it was not normally distributed and involved unpaired continuous variables.
- *Wilcoxon signed-rank test*: For paired analysis of the first and last USD-4D scores in hypothesis 12.

A hypothesis was confirmed when tested significant with *p* ⩽ 0.05.

Patient characteristics were summarized using descriptive statistics: mean and range or median and interquartile range for continuous data, and numbers and percentages for ordinal or dichotomous data. Analyses were conducted using SPSS Statistics 26.^29^

## Results

### Content validity

The online survey was completed by 601 healthcare providers, with 24 excluded as they did not provide palliative care. The final analysis included 577 respondents, predominantly female (88%), with a mean age of 48 years (range: 21–67). Most were nurses (51%). Table 2 summarizes their characteristics.

**Table 2.** Respondents’ characteristics content validity.

Number of respondents	*N*	601	
Gender	Female (%)	509	(85%)
Age	Mean (range)	53.3	(21–67)
Philosophy of life	None	170	(28%)
	Catholic	119	(19%)
	Protestant	224	(34%)
	Islam	4	(1%)
	Spiritual	75	(7%)
	Atheist	5	(1%)
	Other	4	(1%)
Practicing religion	Yes *N* (%)	252	(42%)
Thinking about social issues in daily life	Yes *N* (%)	556	(93%)
Thinking about spiritual issues in daily life	Yes *N* (%)	555	(92%)
Occupation	Nurse assistants	40	(7%)
	Nurses	316	(53%)
	General practitioner	22	(4%)
	Physician	6	(1%)
	Medical specialist	13	(2%)
	Social worker	11	(2%)
	Chaplain	137	(23%)
	Volunteer	18	(3%)
	Coordination	17	(3%)
	Other^a^	21	(3%)
Experience	Years (range)	23.5	(0.5–49)
Additional palliative care courses/education^b^	None	10	(2%)
	Yes, training within basic education	56	(9%)
	Yes, one time training	83	(14%)
	Yes, periodical training	342	(57%)
	Yes, post-HBO for registered nurses	169	(28%)
	Other	145	(24%)
Setting	Home care	177	(29%)
	Hospital	126	(21%)
	Nursing home	127	(21%)
	Hospice	173	(29%)
Palliative care	No	24	(4%)
	Yes, generalist	200	(33%)
	Yes, specialist	377	(63%)

a Among others: dietician, physical therapist, musical therapist.

b Percentages add up to >100% since respondents could give more than 1 answer.

The socio-spiritual reflection in the USD-4D is recognized by 51%–84% of the respondents. Only the item “My thoughts about the end of life, give me peace of mind” is considered either socio-spiritual (51%) or spiritual (48%).

**Figure 4.**
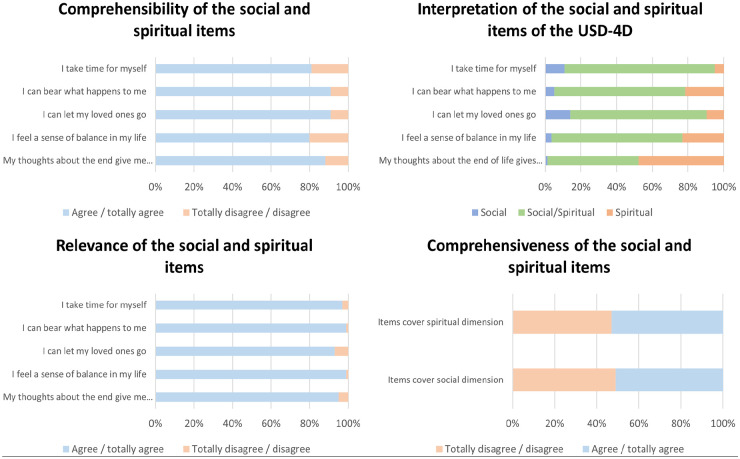
The results for the comprehensibility, relevance and comprehensiveness of the socio-spiritual items.

Figure 4 summarizes results for comprehensibility, relevance, and comprehensiveness.

Comprehensibility of the USD-4D items was excellent, with I-CVI scores between 0.80 and 0.91. Relevance scored even higher, with I-CVI values ranging from 93% to 99%.

**Figure 5.**
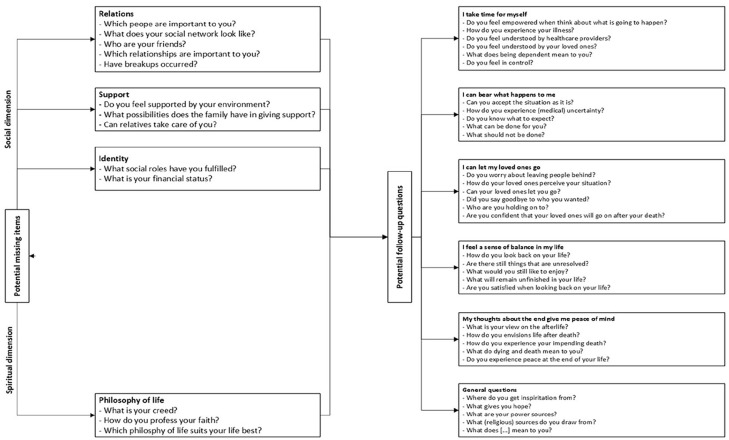
Potential missing items and potential follow-up questions.

Approximately 51% of respondents rated the social dimension as comprehensive, and 53% rated the existential dimension similarly. However, half indicated that some items were missing for full coverage of these dimensions. Qualitative analysis of open-ended responses identified two themes: questions related to medical history and follow-up on the Diamond Model’s polarities (Figure 5). While these are important, they fall outside the USD-4D’s goal.

Overall, the content validity of the USD-4D was confirmed.

#### Construct validity

A total of 897 patients (53% female, aged 68–80) completed at least one USD-4D across various care settings. Most patients had a cancer diagnosis (82%) and received hospice care (85%). Table 3 summarizes their characteristics.

**Table 3.** Respondents’ characteristics construct validity.

	Sympal (hospice setting)		MuSt-PC (all settings)		Total	
Patients, *N* (%)	676	(100.0)	221	(100.0)	897	(100.0)
Age, *N* (%)						
⩽29 years	8	(1.2)	0	(0.0)	8	(.9)
30–45 years	13	(1.9)	8	(3.6)	21	(2.3)
46–67 years	188	(27.8)	86	(38.9)	274	(30.5)
68–80 years	236	(34.9)	80	(36.2)	316	(35.2)
⩾81 years	225	(33.3)	44	(19.9)	269	(30.0)
Missing (%)	6	(.9)	3	(1.4)	9	(1.0)
Gender female—*N* (%)	374	(55.3)	99	(44.8)	473	(52.7)
Missing (%)	7	(1.0)	3	(1.4)	10	(1.1)
Philosophy of life, *N* (%)						
None	56	(8.3)	85	(38.5)	141	(15.7)
Religious	125	(18.5)	109	(49.3)	234	(26.1)
Spiritual	39	(5.8)	19	(8.6)	58	(6.5)
Non-practising	167	(24.7)	0	(0.0)	167	(18.6)
Unknown^a^	283	(41.9)	0	(0.0)	283	(31.5)
Missing (%)	6	(.9)	8	(3.6)	14	(1.2)
Diagnosis, *N* (%)						
Cancer	535	(79.1)	199^b^	(90.0)	734	(81.8)
Organ failure	48	(7.1)	14	(6.3)	62	(6.9)
Cancer + organ failure	26	(3.8)	9	(4.1)	38	(4.2)
Missing (%)	67	(9.9)	0	(0.0)	67	(7.5)
Performance score, *N* (%)						
Restricted activity	74	(10.9)	99	(44.8)	173	(19.3)
<50% of the day in bed	233	(34.5)	40	(18.1)	273	(30.4)
>50% of the day in bed	263	(38.9)	62	(28.1)	325	(36.2)
Completely disabled	56	(8.3)	14	(6.3)	70	(7.8)
Missing (%)	50	(7.4)	6	(2.7)	56	(6.2)
Location of care, *N* (%)						
At home	0	(0.0)	97	(43.9)	97	(10.8)
Hospital	0	(0.0)	31	(14.0)	31	(3.5)
Nursing home	0	(0.0)	2	(.9)	2	(.2)
Hospice	676	(100.0)	84	(38.0)	760	(84.7)
Other	0	(0.0)	3	(1.4)	3	(.3)
Missing (%)	0	(0.0)	4	(1.8)	4	(.4)

a In the hospice database this category reflects patients not knowing how to reflect on their philosophy of life.

b Participants in the MuSt-PC study could indicate more than 1 disease; MuSt-PC and total numbers add up to >100%.

All hypotheses except one (hypothesis 12) were statistically significant. With 75%–100% of hypotheses confirmed per item, the construct validity of the socio-spiritual items was established (Tables 4 and 5).

**Table 4.** Hypothesis testing of the socio-spiritual items of the USD-4D—pain, anxiety, depressed mood, and time to death.

		*N*	USD-4D score ⩾6 *N* (%)		*p*-Value
*I take time for myself*					
1	Severe pain (⩾6)	131	30	(23)	0.002^†^
	No pain (0)	327	37	(11)	
2	Severe anxiety (⩾6)	91	33	(36)	<0.001^†^
	No anxiety (0)	501	60	(12)	
3	Severe depressed mood (⩾6)	97	34	(35)	<0.001^†^
	No depressed mood (0)	442	41	(9)	
*I can bear what happens to me*					
5	Severe pain (⩾6)	134	38	(28)	<0.001^†^
	No pain (0)	331	33	(10)	
6	Severe anxiety (⩾6)	90	35	(39)	<0.001^†^
	No anxiety (0)	508	51	(10)	
7	Severe depressed mood (⩾6)	99	45	(46)	<0.001^†^
	No depressed mood (0)	446	27	(6)	
*I can let my loved ones go*					
9	Severe anxiety (⩾6)	91	58	(64)	<0.001^†^
	No anxiety (0)	490	207	(42)	
10	Severe depressed mood (⩾6)	102	65	(64)	<0.001^†^
	No depressed mood (0)	426	180	(42)	
12	USD-4D ⩽2 weeks before death	163	63	(39)	0.076^‡^
	First USD-4D	163	72	(44)	
*I feel a sense of balance in my life*					
13	Severe pain (⩾6)	127	40	(32)	<0.001^†^
	No pain (0)	316	39	(12)	
14	Severe anxiety (⩾6)	88	39	(44)	<0.001^†^
	No anxiety (0)	483	60	(12)	
15	Severe depressed mood (⩾6)	98	45	(46)	<0.001^†^
	No depressed mood (0)	423	44	(10)	
*The thought about the end of life gives me peace of mind*					
17	Severe anxiety (⩾6)	57	34	(60)	<0.001^†^
	No anxiety (0)	290	95	(33)	
18	Severe depressed mood (⩾6)	66	43	(65)	<0.001^†^
	No depressed mood (0)	252	78	(31)	

**Table 5.** Hypothesis testing of the socio-spiritual items of the USD-4D—well-being.

			Median well-being score [IQR]	*p*-Value*
4	I take time for myself	Score ⩾6	5 [4–7]	<0.001
		Score 0–5	3 [0–5]	
8	I can bear what happens to me	Score ⩾6	5 [4–7]	<0.001
		Score 0–5	3 [1–5]	
11	I can let my loved ones go	Score ⩾6	4 [2–5]	0.041
		Score 0–5	4 [1–5]	
16	I feel a sense of balance in my life	Score ⩾6	5 [3–6]	<0.001
		Score 0–5	3 [1–5]	
19	The thought about the end of life gives me peace of mind	Score ⩾6	4 [2–5]	0.037
		Score 0–5	4 [1–5]	

IQR: interquartile range.

* Mann–Whitney *U*.

## Discussion

This study assessed the content and construct validity of the socio-spiritual items in the USD-4D. From healthcare providers’ perspectives, the items demonstrated strong content validity, with I-CVI scores of 0.8–0.93 for comprehensibility and relevance. Most healthcare providers recognized these items as addressing both social and spiritual dimensions, although about 50% felt certain items were missing. However, the suggested additions were outside the USD-4D’s purpose of monitoring concern severity over time. At least 75% of hypotheses per item were confirmed, with 18 of 19 hypotheses validated. Thus, the USD-4D’s content and construct validity are affirmed.

The USD-4D, now a validated multidimensional PROM, can be used to effectively monitor patients’ symptoms and needs and facilitate patient-driven dialogue, supporting care tailored to their values and wishes. Content validity is particularly crucial for multidimensional PROMs, like the USD-4D, that do not focus on a single construct.^31^ Unlike PROMs such as the SDI,^10^ FACIT-SP12,^11^ and SNAP,^12^ which categorize items strictly within spiritual or social domains, the USD-4D adopts a flexible approach, allowing patients to articulate needs without rigid definitions. This flexibility aligns with research indicating that patients often use overlapping language for social and spiritual needs, promoting a holistic understanding.^9^

With only five socio-spiritual items, the USD-4D is not exhaustive but serves as a starting point to identify care needs through patient-driven dialogue. These items, derived from the Diamond Model’s polarities, are broad in scope to accommodate diverse interpretations, requiring healthcare providers to skillfully facilitate discussions to clarify and address patient concerns.^17^ Studies have shown that patients’ interpretations align well with the intended polarities, supporting the items’ validity while maintaining their adaptability.

The USD-4D is not designed to measure a single predefined construct, posing challenges for assessing construct validity. Despite this, combining theoretical frameworks and expert insights enabled hypothesis testing, validating the socio-spiritual items. The study deliberately avoided evaluating convergent validity due to the subjective and multifaceted nature of the social and spiritual dimensions, which complicates correlations with other instruments. This decision highlights the USD-4D’s unique role in capturing nuanced patient perspectives within these dimensions.

### Strengths and limitations

This study’s strength in assessing content validity lies in the large response rate from HCPs and the representative distribution of respondents, reflecting clinical practice where the USD-4D is used. Generalist and specialist HCPs, especially nurses who primarily use the USD-4D in palliative care, were well represented. A multidisciplinary research team, comprising nurses, a chaplain, and an ethicist, enhanced data analysis and ensured conclusions aligned with clinical practice.

Some limitations should be considered. Data were collected exclusively through surveys, without focus groups as recommended by COSMIN methodology. However, the large sample size and consensus in responses suggest surveys were sufficient for evaluating content validity. Participants were mainly female (88%), which reflects national numbers and thus will not skew the validity measure.^32^ Additionally, 42% of respondents practiced religion, differing from national averages, but sensitivity analysis indicated no effect on results. Moreover, we did not study participants’ interpretation of spirituality. Physician, medical specialist, and social worker participation was lower, but primary end-users were well represented.

This study is the first to assess the construct validity of the USD-4D’s socio-spiritual items among Dutch palliative patients across care settings. Hypotheses were formed through literature and interprofessional collaboration, enhancing their relevance. Despite limited data from hospital and home care settings, results were robust for hospice settings, with 75% of hypotheses confirmed. The unconfirmed hypothesis on “I can let go of my loved ones” may reflect a lack of data from patients near death, as they typically do not complete the USD-4D in their final days.

### Implications for clinical practice

The validated content and construct of the USD-4D’s socio-spiritual items support its use as a reliable PROM in clinical practice. It is important to note that while these findings confirm the items’ accuracy and alignment with relevant hypotheses, they do not constitute formal proof or evidence of causality. Compared to other international PROMs focusing on the social and spiritual dimensions, the USD-4D has proven to be a clinically feasible PROM to monitor and facilitate dialogue on patients’ multidimensional symptoms and needs and foster holistic palliative care. After culturally sensitive translation and cross-cultural validation the USD-4D warrants international use.

For meaningful interpretation, ongoing dialogue between patients and healthcare providers is essential. The USD-4D should be integrated into clinical palliative care as more than a tool, becoming part of multiprofessional workflows and supported by continuous skill development for healthcare providers.

### Implications for future research

This study focused on an autochthonous Dutch palliative care population. To ensure the USD-4D’s effectiveness in other cultures, future research should address cross-cultural validity. Further development is also needed for its use outside palliative care or with cognitively impaired patients.

Validation is an ongoing process, requiring continuous refinement as understanding of the constructs and empirical evidence grows. Future studies should further validate the USD-4D in diverse contexts, settings, and populations while also re-evaluating the underlying theories supporting the instrument.^12^

## Conclusion

This study confirmed the content validity from healthcare providers’ perspectives and construct validity of the USD-4D’s socio-spiritual items, showing it effectively measures its intended constructs. By reliably monitoring multidimensional symptoms and needs, the USD-4D supports patient-centered palliative care. Furthermore, it shows how clinically feasible PROMs can be structurally used. It also highlights the need for patient-driven research into socio-spiritual well-being, emphasizing tools sensitive to the complex needs of patients facing a life-limiting illness without being too elaborate.

## Appendix 1

### The Utrecht Symptom Diary—4 Dimensional

**The scores are provided by:** 0 patient   0 loved one/relative   0 caregiver

**I have**

**The scores are provided by:** 0 patient   0 loved one/relative   0 caregiver

**I take time for myself**
